# Age-related decrease of miRNA-92a levels in human CD8^+ ^T-cells correlates with a reduction of naïve T lymphocytes

**DOI:** 10.1186/1742-4933-8-11

**Published:** 2011-11-15

**Authors:** Michiyo Ohyashiki, Junko H Ohyashiki, Ayako Hirota, Chiaki Kobayashi, Kazuma Ohyashiki

**Affiliations:** 1 Department of Hematology, Tokyo Medical University, Tokyo, Japan; 2 Institute of Medical Science, Tokyo Medical University, Tokyo, Japan

**Keywords:** cellular miR-92a, naïve T lymphocytes, ageing

## Abstract

MicroRNA (miR)-17-92a expression plays a crucial role in lymphocyte ontogeny. We therefore set out to determine miR-92a expression levels in peripheral blood lymphocytes from healthy subjects to ascertain any association between these levels and ageing. We found a positive correlation between the miR-92a expression level and the percentages of RO-CD8^+^CD27^+ ^(*P *= 0.0046) and CD3^+^CD8^+^CD62L^+ ^(*P *= 0.0011). This suggests that the majority of miR-92a of CD8^+ ^T cells is derived from naïve cells, and the miR-92a expression level in CD8^+ ^T cells declines progressively with age. These results indicate that the age-related attrition of naïve T cells is linked to a reduction of miR-92a in human T -lymphocytes. Therefore, we should careful attention when evaluating human miRNA levels in T lymphocytes to use normal control values.

## Background

MicroRNAs (miRNAs) consist of short noncoding RNA molecules of approximately 18-22 nucleotides that regulate post-transcriptional gene expression by degradation or repression of mRNA molecules. The miR-17-92a cluster is known to be a regulator of the immune system and is critical for lymphoid cellular development and tumorigenesis in lymphoid tissue [1-3]. Most knowledge of the miR-17-92a cluster in normal and abnormal conditions of the lymphoid system is based on mouse experiments. The absence of miR-17-92a up-regulates *BIM*, which inhibits B-cell development at the pro-B to pre-B transition [4]. High expression of miR-17-92a in transgenic mice leads to expansion of the CD4^+ ^lymphocyte pool, and the naïve CD4^+ ^cells show recruitment and activation of phosphatidylinositol-3-OH-kinase via suppression of *PTEN *[3,5]. This suggests that the accumulation of activated CD4^+ ^T cells by higher mir-17-92a expression leads to a breakdown of T-cell tolerance in the periphery and may promote B-cell activation, germinal centre reaction and autoantibody generation.

In humans, however, senescence of lymphocytes *in vivo *occurs through maturation of antigen-stimulation, and thereby subset fractions of lymphocytes gradually change with age; for example, an increase of memory T cells takes place by progressive naïve T-cell reduction. Although miR-17-92a expression is crucial for lymphocyte development, only limited reports on *in vivo *human lymphocyte senescence exist. We therefore set out to determine miR-92a levels in peripheral blood lymphocytes obtained from healthy individuals to ascertain the possible association between the expression level of miR-17-92a and ageing.

## Results and Discussion

**Figure 1:**
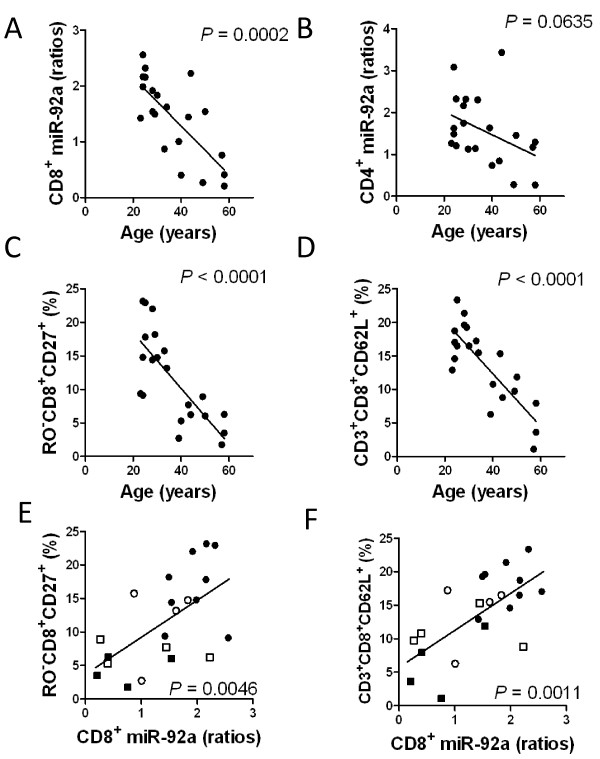
**Correlation between T-lymphocyte miR-92a level and age**. The CD8^+ ^T-lymphocyte miR-92a level significantly decreased with age (A), while CD4^+ ^lymphocytes only showed a tendency for a decrease of miR-92a level with age (B). A significant decrease in the percentage of RO^-^CD8^+^CD27^+ ^fraction (C) or CD3^+^CD8^+^CD62L^+ ^fraction (D) with age is evident. There is a significant positive correlation between CD8^+ ^miR-92a level (*x *axis) and the percentage of RO^-^CD8^+^CD27^+ ^fraction (E) and CD3^+^CD8^+^CD62L^+ ^fraction (F) (*y *axis), which tends to down-regulate with age (closed circles = 20 to 29 years; open circles = 30 to 39 years; open squares = aged 40 to 49 years; closed squares = older than 50 years).

The miR-92a in separated CD8^+ ^T cells decreased significantly with age (*P *= 0.0002) (Figure 1-A), and miR-92a in CD4^+ ^cells tended to decrease with age (*P *= 0.0635) (Figure 1-B) (Additional file 1 Table S1). The percentage of RO^-^CD8^+^CD27^+ ^(Figure 1-C) and CD3^+^CD8^+^CD62L^+ ^fractions (Figure 1-D) also significantly decreased with age (*P *< 0.0001 and *P *< 0.0001, respectively) (Additional file 1 Table S1). Although the RO^-^CD8^+^CD27^+ ^fraction in this study was not the strictly a naïve CD8 population, we considered it to represent a major proportion of naïve CD8^+ ^T cells. The miR-92a in CD4^+ ^T cells did not show any significant relationship with the lymphocyte subset fraction. In contrast, the miR-92a level in CD8^+ ^T cells was significantly correlated with percentages of the cell fraction of RO^-^CD8^+^CD27^+ ^cells (*P *= 0.0046) (Figure 1-E) and CD3^+^CD8^+^CD62L^+ ^cells (*P *= 0.0011) (Figure 1-F) (Additional file 2 Table S2). This suggests that the majority of miR-92a in CD8^+ ^T-cells may originate from naïve cytotoxic T cells, in accordance with the report by Salaun *et al*. [6]. By both direct cloning and real-time PCR methods, Wu *et al*. also demonstrated that antigen-specific naïve CD8^+ ^cells obtained from mice had elevated miR-92a [7]. Therefore, it is likely that both reduced miR-17-92a cluster expression and down-regulation of the naïve cytotoxic T-cell fraction with age may lead to reduced amounts of miR-92a derived from naïve cytotoxic T cells (Additional file 3 Figure S1-A to D). In CD4^+ ^cells, we observed an age-related increase of the ratio of central memory to naïve CD4^+ ^cells, without any correlation to CD4^+ ^miR-92a levels (Additional file 3 Figure S1-E and F).

It has been shown that the diminished responsiveness of naïve CD8^+ ^cells in older humans coincides with a progressive loss of naïve T-cell receptor (TCR) repertoire diversity, and antigens exposure through infection in humans younger than 50 years selects specific clones from the TCR repertoire and generally induces protective and long-lived memory T-cell responses [8]. Therefore, we speculated that down-regulation of miR-92a in naïve CD8^+ ^T cells with ageing may be linked to progressive loss of naïve TCR repertoire diversity due to antigen exposure, including latent viral infection [8]. However, we do not have any direct evidence for the relationship between antigenic stress and infection status, including cytomegalovirus (CMV) infection [9,10], that may be linked to the progressive decline of miR-92a expression in naïve cytotoxic T cells.

This is a first report that an age-related decline of miRNA levels is correlated with a reduced population of naïve CD8^+ ^T lymphocytes in normal human subjects. Recently, Salaun *et al*. [6] demonstrated that the miR-17-92a cluster is one of the most highly expressed mRNAs in isolated human naïve T cells and its expression tends to be down-regulated in more differentiated cells. We also noticed down-regulation of miR-92a in both CD4^+ ^and CD8^+ ^lymphocytes with age, in accordance with the report by Hackl, *et al*. [11] that showed miR-17, miR-19b, miR-20a, and miR-106a were down-regulated in human cells, including CD8^+ ^lymphocytes, in an aging population. Down-regulation of the miR-17-92a cluster in human fibroblasts has also been reported in age-related conditions, such as stress-induced senescence or low-level irradiation [12,13].

## Conclusions

In conclusion, our results suggest that the miR-92a level may represent attrition of human naïve CD8^+ ^T cells, possibly due to apoptosis of naïve T cells. Although the number of subjects in this study was too small to provide a definitive conclusion, down-regulation of the miR-92a level may indicate exhaustion of naïve T-cells due to alteration of the immunologic condition with ageing, especially in individuals older than 60 years. Therefore, it would be prudent to pay careful attention in interpreting human miRNAs levels in healthy controls since the expression levels of some miRNAs, such as miR-17-92a in lymphocytes, show age-related alterations.

## Methods

### Samples

We separated lymphocytes from 21 healthy volunteers, aged 23 to 58 years (13 men and 8 women), for surface marker and miR-92a level analyses. This study was approved by the institutional review board of Tokyo Medical University (no. 930: approved on June 24, 2008), and written informed consent was obtained from all participants prior to collection of specimens according to the Declaration of Helsinki. For lymphocyte separation and cell surface marker analysis,

### Phenotyping

Heparinized whole blood was obtained and mononuclear cells were separated by a gradient method. Immunophenotyping was done with flow cytometry by using antibodies against the following antigens: CD3, CD4, CD8, CD16, CD45, CD56, CD57, HLA-DR, CD27, CD62L, CD45RO, CD19, CD20, TCR-α/β and TCR-γ/δ (Beckman Coulter, Miami, Florida, USA). The analyses were performed with a three-color flow cytometer (EPICS XL, Beckman Coulter).

### Lymphocyte separation

The CD4^+ ^or CD8^+ ^T-cell fractions were separated with an isolation kit for humans (Miltenyi Biotec, Bergisch Gladbach, Germany) and AutoMACS Pro Separator (Miltenyi Biotec), according to the supplier's instruction, and stored at -80°C until utilization. The isolated cell purification was >95%.

### MiR-92a quantitative RT-PCR

Total RNA in cells was isolated with a miRNeasy Mini Kit (Qiagen, Germantown, Maryland, USA) as reported previously, and 10 ng RNA was used for this study. The miR-specific stem-loop primers hsa-miR-92a (000431; Applied Biosystems, Foster City, California, USA) and RNU6B (001093; Applied Biosystems), were used in the study. The expression of miR-92a was calculated using 2¯^ΔΔ*Ct *^methods, and mean cycle threshold (*C^t^*) values for all miRNAs were quantified using sequence detection system software (SDS, version 1.02; Applied Biosystems). We used total RNA obtained from separated lymphocytes of healthy volunteers, and the miR-92a expression levels in specimens were expressed as 'ratios' [14].

### Statistical methods

GraphPad 5.0 software (GraphPad Software Inc, San Diego, CA, USA) was used for statistical analysis. We used linear regression analysis and obtained *P *values from the 'Slope significantly non-zero' and *R*^2 ^value from the 'Goodness of Fit' for correlation between two independent parameters. *P *values less than 0.05 were considered to indicate statistically significant differences.

## Competing interests

The authors declare that they have no competing interests.

## Authors' contributions

MO, KO and JHO designed the study, analyzed the data, and wrote the manuscript; CK and MO performed the quantitative PCR analysis of miRNA; KO performed the statistical analysis; AY performed the surface marker analysis and separation of lymphocytes; JHO and KO contributed to the interpretation of the data. All authors read and approved the final manuscript.

## Supplementary Material

Additional file 1 **Correlation between age and percentage of lymphocyte fraction or miR-92a expression level**. Click here for file

Additional file 2 **Correlation between miR-92a and percentage of lymphocyte fraction**. Click here for file

Additional file 3 **Correlation between T-lymphocyte subset and age**. An increase in the ratios of central memory to naïve CD8^+ ^(*P *= 0.0663) (A), effector memory to naïve CD8^+ ^(*P *= 0.0527) (C), and central memory to naïve CD4*^+ ^*(*P *= 0.0096) (E) with age is notable. The miR-92a level in CD8^+ ^T-cells is negatively correlated with the ratio of central memory to naïve CD8^+ ^cells (*P *= 0.0016) or effector memory/naïve CD8^+ ^cells (*P *= 0.0073). By contrast, the miR-92a level in CD4^+ ^T-cells is not correlated with the ratio of central memory to naïve CD4^+ ^cells (*P *= 0.0925) (F). Click here for file
