# Supplementary figures and images for: Age-related decrease of miRNA-92a levels in human CD8+ T-cells correlates with a reduction of naïve T lymphocytes
Source: Immun Ageing. 2011 Nov 15;8:11. doi: 10.1186/1742-4933-8-11 (PMC3225295; doi:10.1186/1742-4933-8-11)

## Slide 1
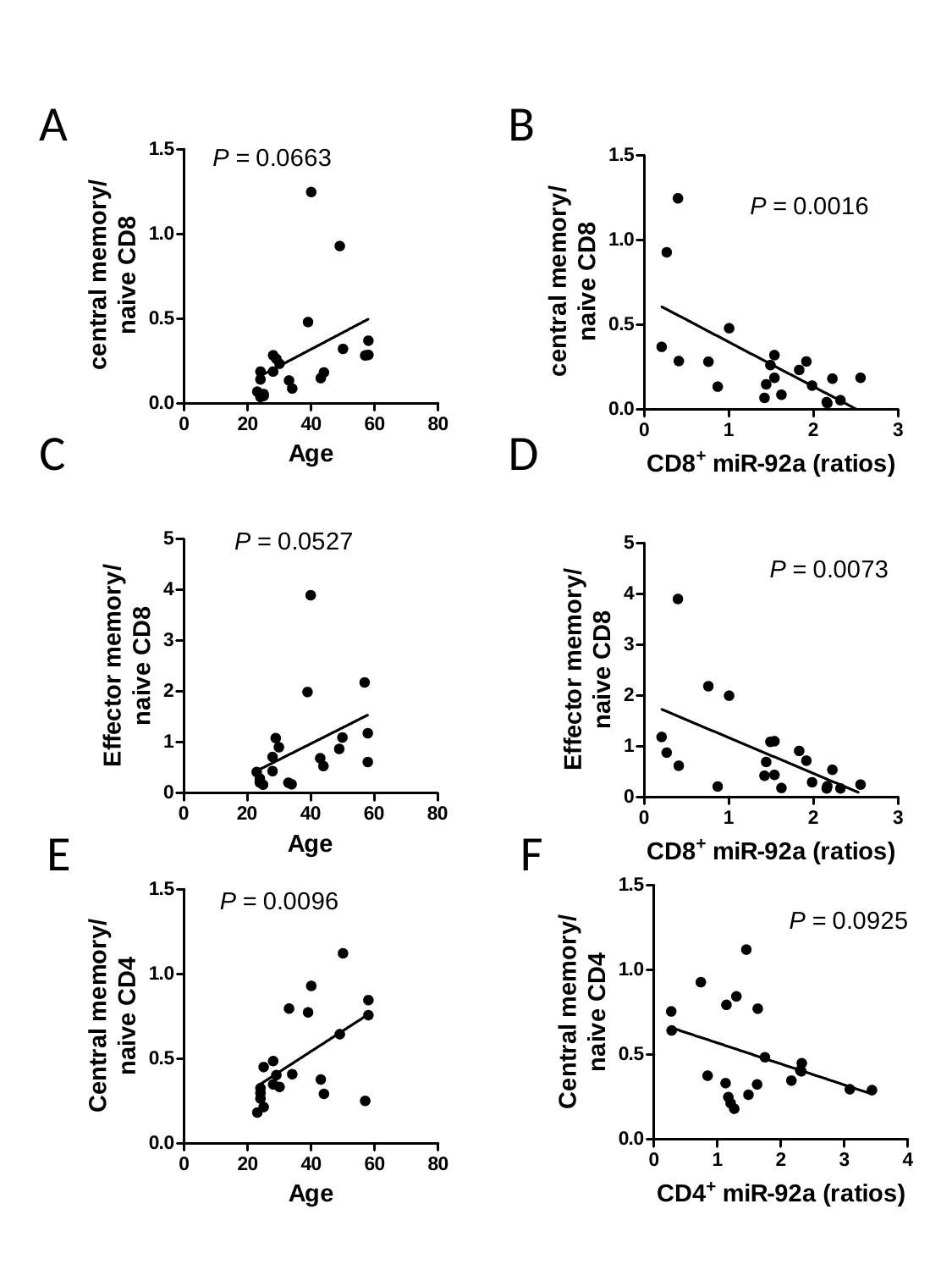

A
B
C
D
E
F

Supplement: Additional file 3 — Correlation between T-lymphocyte subset and age. An increase in the ratios of central memory to naïve CD8+ (P = 0.0663) (A), effector memory to naïve CD8+ (P = 0.0527) (C), and central memory to naïve CD4+ (P = 0.0096) (E) with age is notable. The miR-92a level in CD8+ T-cells is negatively correlated with the ratio of central memory to naïve CD8+ cells (P = 0.0016) or effector memory/naïve CD8+ cells (P = 0.0073). By contrast, the miR-92a level in CD4+ T-cells is not correlated with the ratio of central memory to naïve CD4+ cells (P = 0.0925) (F). [file 1742-4933-8-11-S3.PPTX]
