# Supplementary material for: Age-related decrease of miRNA-92a levels in human CD8+ T-cells correlates with a reduction of naïve T lymphocytes
Source: Immun Ageing. 2011 Nov 15;8:11. doi: 10.1186/1742-4933-8-11 (PMC3225295; doi:10.1186/1742-4933-8-11)
Supplement: Supplementary file 2 [file 1742-4933-8-11-S1.DOC]

		R^2 of Goodness of fit	P value of slope significance
MiR-92a expression			
	$CD4^+$ miR-92a level	0.1697	0.0635
	$CD8^+$ miR-92a level	0.536	0.0002
Lymphocyte subset fraction			
	CD45 gating $CD8^+$ (%)	0.277	0.0142
	CD45 gating TCR-γδ (%)	0.09675	0.1699
	CD45 gating TCR-αβ (%)	0.02898	0.4606
	$CD3^+CD8^+$ TCR-γδ (%)	0.02371	0.5051
	$CD3^+CD8^+$ TCR-αβ (%)	0.0001715	0.9551
	$CD3^+CD8^+CD57^+$ (%)	1.22E-02	0.6331
	$CD3^+CD8^+CD62L^+$ (%)	0.6646	< 0.0001
	$CD3^+CD8^+$HLA-DR^+ (%)	0.0009812	0.8928
	$RO^+CD8^+CD27^+$ (%)	0.001181	0.8824
	$RO^+CD8^+CD27^-$ (%)	0.007968	0.7004
	$RO^-CD8^+CD27^+$ (%)	0.567	< 0.0001
	CD45 gating $CD4^+$ (%)	0.0121	0.635
	$RO^+CD4^+CD27^+$ (%)	0.1595	0.0729
	$RO^-CD4^+CD27^+$ (%)	0.1175	0.1282
	$CD19^+CD20^+$	0.04656	0.3475
	$CD16^+CD56^+$	0.07476	0.2304
