# Supplementary material for: Age-related decrease of miRNA-92a levels in human CD8+ T-cells correlates with a reduction of naïve T lymphocytes
Source: Immun Ageing. 2011 Nov 15;8:11. doi: 10.1186/1742-4933-8-11 (PMC3225295; doi:10.1186/1742-4933-8-11)
Supplement: Supplementary file 3 [file 1742-4933-8-11-S2.DOC]

		R^2 of Goodness of fit	P value of slope significance
$CD4^+$ miR-92a			
	CD45 gating $CD4^+$ (%)	0.07789	0.2205
	CD45 gating TCR-γδ (%)	0.0001429	0.959
	CD45 gating TCR-αβ (%)	0.0001813	0.9538
	$RO^+CD4^+CD27^+$ (%)	0.0976	0.168
	$RO^-CD4^+CD27^+$ (%)	0.04204	0.3726
$CD8^+$ miR-92a			
	CD45 gating $CD8^+$ (%)	0.4119	0.0017
	CD45 gating TCR-γδ (%)	0.06668	0.2584
	CD45 gating TCR-αβ (%)	0.001967	0.8486
	$CD3^+CD8^+$ TCR-γδ (%)	0.01152	0.6433
	$CD3^+CD8^+$ TCR-αβ (%)	0.03756	0.3999
	$CD3^+CD8^+CD57^+$ (%)	2.51E-04	0.9457
	$CD3^+CD8^+CD62L^+$ (%)	0.4382	0.0011
	$CD3^+CD8^+HLA\text{-}DR^+$ (%)	0.1193	0.1251
	$RO^+CD8^+CD27^+$ (%)	0.1101	0.1418
	$RO^+CD8^+CD27^-$ (%)	0.02938	0.4575
	$RO^-CD8^+CD27^+$ (%)	0.3512	0.0046
